# How does HIV testing modality impact the cascade of care among persons diagnosed with HIV in Ethiopia?

**DOI:** 10.1080/16549716.2021.1933788

**Published:** 2021-08-17

**Authors:** Malin Johansson, Clara Penno, Niclas Winqvist, Fregenet Tesfaye, Per Björkman

**Affiliations:** a Clinical Infection Medicine, Department of Translational Medicine, Lund University, Malmö, Sweden; b Mycobacterial Disease Research Department, Armauer Hansen Research Institute, Addis Ababa, Ethiopia; c Department of Infectious Diseases, Skåne University Hospital, Malmö, Sweden

**Keywords:** HIV, Ethiopia, test modality, community-based testing, cascade of care

## Abstract

**Background:**

Despite scaling up of HIV programmes in sub-Saharan Africa, many people living with HIV (PLHIV) are unaware of their HIV status. New testing modalities, such as community-based testing, can improve test uptake, but it is uncertain whether type of testing modality affects the subsequent cascade of HIV care.

**Objective:**

To compare linkage to care and antiretroviral treatment (ART) outcomes with regard to type of HIV testing modality.

**Methods:**

A retrospective registry-based study was conducted at public ART clinics in an urban uptake area in Central Ethiopia. Persons aged ≥15 years newly diagnosed with HIV in 2015–2018 were eligible for inclusion. Data on patient characteristics and testing modality were analysed for associations with the following outcomes: ART initiation, retention in care at 12 months after starting ART, and viral suppression (<1000 copies/ml, recorded during the first 12 months after ART initiation), using uni- and multivariable analysis. Separate analyses disaggregated by sex were performed.

**Results:**

Among 2885 included PLHIV (median age 32 years, 59% female), 2476 (86%) started ART, 1422/2043 (70%) were retained in care, and 953/1046 (92%) achieved viral suppression. Rates of ART initiation were lower among persons diagnosed through community-based testing (adjusted odds ratio [AOR] 0.44, 95% confidence interval [CI] 0.29–0.66) and among persons diagnosed through provider-initiated testing (AOR 0.65, 95% CI 0.44–0.97) compared with facility-based voluntary counselling and testing. In sex-disaggregated analyses, community-based testing was associated with lower rates of ART initiation among both women and men (AOR 0.47, 95% CI 0.27–0.82; AOR 0.39, 95% CI 0.19–0.78, respectively). No differences were found for retention in care or viral suppression with regard to test modality.

**Conclusion:**

Type of HIV testing modality was associated with likelihood of ART initiation, but not with subsequent treatment outcomes among persons starting ART.

## Background

Despite the expansion of HIV care in sub-Saharan Africa during the recent decade, nearly half of people living with HIV (PLHIV) are estimated to be unaware of their HIV status [1,2]. In order to facilitate access to HIV testing for populations that are not reached by facility-based testing services, different types of community-based strategy for HIV testing have been introduced. Community-based testing can occur as campaigns, commonly targeting high-risk populations, as well as testing offered in workplaces or home-based testing programmes (which can also be performed as self-testing) [3].

Community-based testing modalities have been shown to increase HIV case-finding. A large meta-analysis including 126 studies performed in sub-Saharan Africa showed that community-based testing modalities were more likely to reach men and young adults than facility-based testing, and that PLHIV identified through non-facility-based testing modalities had higher CD4 counts at diagnosis [3]. However, it has been reported that persons diagnosed through community-based testing are at elevated risk of inadequate linkage to care. Studies from Mozambique, Tanzania and South Africa have shown community-based testing to be associated with lower rates of linkage, implying the need of interventions to facilitate this procedure [4–6]. Furthermore, it is possible that completion of the subsequent components of the cascade of HIV care, initiation of antiretroviral therapy (ART), retention in care and achievement of viral suppression may differ with regard to testing modality, but data on these issues are hitherto limited.

In Ethiopia, the national HIV prevalence is estimated to be 1.2% [7], with HIV/AIDS among the leading causes of death [8]. Free HIV testing, as well as ART, has been available through the public health sector since 2005, with rapid scale-up during the last decade. Currently, over 70% of people diagnosed with HIV are estimated to have initiated ART [9]. Community-based testing options, including campaigns, workplace testing and mobile VCT, have gradually been introduced in Ethiopia to increase access to testing [10] but it is unknown how outcomes of HIV care differ with regard to testing modality.

In this study, we have investigated associations between HIV testing modality and different components of the cascade of HIV care for persons newly diagnosed with HIV in an urban uptake area in Central Ethiopia.

## Methods

### Study setting and design

Participants in this retrospective study were identified at clinics providing HIV care at public health facilities (three hospitals and five health centres) in and around the city of Adama, Ethiopia. HIV prevalence among pregnant women in Adama has been estimated at 9.3% [11], which is among the highest reported in the country. The uptake area is urban/semi-urban and has a population of around 600,000 inhabitants.

Individuals newly diagnosed with HIV infection were identified from registers kept at the study sites. All persons registered from June 2015 to September 2018 were eligible for inclusion. Persons with no registered information on test modality, with registered start of ART before HIV diagnosis, and/or age ≤15 years were excluded.

### Data collection

Data were collected from registers and medical records kept at the ART clinics (September–November 2019), and included baseline sociodemographic and clinical characteristics, as well as data on ART initiation, follow-up visits and viral load (VL) results. The information obtained from the registers was cross-checked with medical records for 5% of randomly selected participants (error rate <3%).

Type of test modality was classified as community-based, voluntary counselling and testing (VCT), provider-initiated counselling and testing (PICT), or ‘other’. Community-based testing included all types of testing performed at sites other than health facilities. In most cases, community-based testing was conducted by non-governmental organizations (NGOs). In the uptake area of this study, different national NGOs conduct testing campaigns regularly. Most community-based testing (52%) was performed by the NGO ‘Organization for Social Services for AIDS’ (OSSA), a nationwide organization providing HIV care and support for PLHIV. These testing campaigns usually occurred at mobile temporary clinics, with particular focus on key populations.

For facility-based test modalities, VCT was defined as client-initiated testing at designated clinics. PICT included all opt-out facility-based testing (offered for patients with tuberculosis [TB] or sexually transmitted infections, women attending antenatal care [ANC] and persons seeking outpatient care meeting predefined criteria). The category ‘other’ included persons tested at private clinics, persons tested through contact tracing, and persons who had been transferred from other public clinics after receiving their first positive HIV test result.

Three separate outcomes were investigated: (1) ART initiation (for all included participants); (2) retention in care at 12 months after ART initiation (for participants who started ART) and (3) virological suppression recorded during the first 12 months of ART (for participants with registered ART duration of >6 months).

ART initiation was defined as having a registered date for starting ART (with a date not preceding the date of HIV diagnosis). Retention in care was defined as remaining in care at 12 months after the date of ART initiation, with no registered loss-to-follow-up (LTFU; >90 days of missing planned clinic visits) during this period. For this analysis, women diagnosed during ANC were excluded (since management is routinely transferred to ANC clinics during pregnancy and post-partum), as well as participants with registered transfer of care. Virological suppression was defined as ≥1 registered VL result <1000 copies/ml obtained during the first 12 months after ART initiation, with no recorded VL result ≥1000 copies/ml during this period. Participants without registered VL results were excluded from this analysis.

### Statistical analysis

The three study outcomes were compared between participants with regard to test modality and the following baseline characteristics: age, sex, body mass index (BMI), concomitant active TB and clinical stage (World Health Organization [WHO] disease stage, I–IV). All of these characteristics have been shown to be associated with outcomes of HIV care in earlier studies [12–16]. All characteristics were managed as categorical variables. Age was stratified into three groups: 16–25, 26–35 and ≥36 years, respectively. BMI <18.5 was used to define underweight. WHO clinical stage was separated as early (I–II) or late (III–IV).

Associations between baseline variables including type of test modality and the respective outcomes were tested in univariate analysis with χ^2^-test. Odds ratios (OR) were calculated for each variable using categories associated with better outcomes (based on previous findings [12–16]) for reference (age ≥36 years, female sex, BMI >18.5, absence of concomitant TB, WHO stage I–II). Variables with p < 0.2 in univariate analysis were subsequently entered into multivariable logistic regression analysis. In the multivariable analysis, forward selection was used to determine factors independently associated with the study outcomes. Separate sex-disaggregated analyses were performed. p < 0.05 was used to define statistical significance.

### Ethical approval

Ethical approval was obtained from the Armauer Hansen Research Institute Ethics Review Committee and from the Oromia Regional Health Bureau Ethical Review Board, Addis Ababa, Ethiopia.

## Results

### Participant characteristics

**Figure 1.**
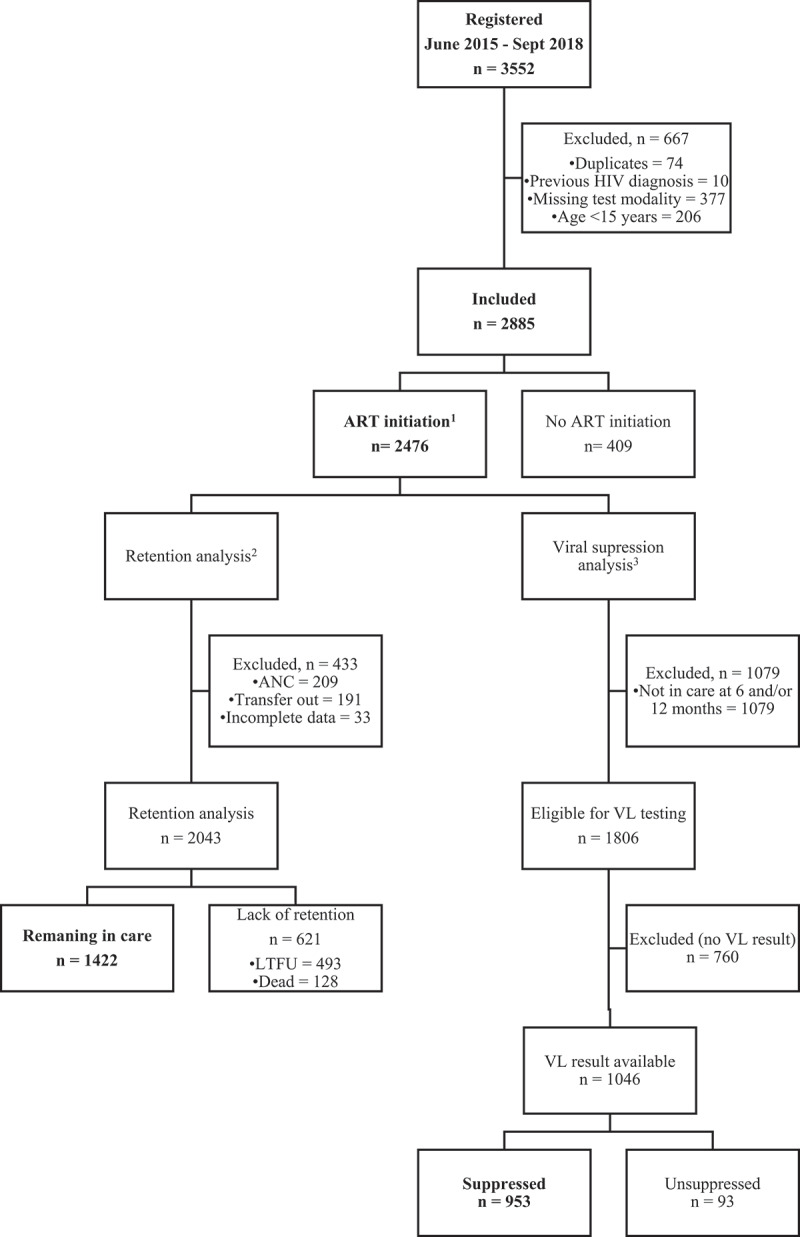
Flowchart of inclusion of participants in the different components of the study

Abbreviations: ART – antiretroviral therapy. ANC – antenatal care. VL – viral load. Suppressed: ≥1 VL <1000 copies/ml, with no recorded VL ≥ 1000 copies/ml. Unsuppressed: ≥1 VL ≥1000 copies/ml. ^1^For analysis of ART initiation, all participants were included. ^2^For analysis of retention in care, the following exclusion criteria were applied: diagnosed during antenatal care (ANC); transfer of care to a different clinic (transfer out); incomplete follow-up data. ^3^For analysis of viral load testing, participants with no registered viral load result were excluded.

During the study period, 3552 newly diagnosed PLHIV were registered at the study facilities, 2885 of whom were included. A flowchart of inclusion of participants for the three study components is presented in Figure 1.

Baseline characteristics are shown in Table 1. The following types of test modality were registered: community-based 583 (20%), VCT 561 (19%), PICT 1164 (40%), and 'other' 577 (20%).

**Table 1.** Characteristics of 2885 adults and adolescents newly diagnosed with HIV at Ethiopian health facilities with regard to type of test modality

	Total n (%^1^)	VCT n (%)	Community n (%)	PICT n (%)	Other n (%)
Total n (%)	2885 (100)	561 (19)	583 (20)	1164 (40)	577 (20)
**Age (years)**					
*Median [IQR]*	*32 [27–40]*	*33 [27–40]*	*32 [27–38]*	*30 [26–40]*	*35 [28–40]*
16–25	566 (20)	98 (18)	120 (21)	268 (24)	80 (15)
26–35	1225 (43)	249 (45)	263 (46)	482 (42)	231 (42)
≥36	1035 (37)	209 (38)	195 (34)	392 (34)	239 (44)
Missing	59 (2)	5 (1)	5 (1)	22 (2)	27 (5)
**Gender**					
Female	1704 (59)	326 (58)	335 (57)	732 (63)	311 (54)
Male	1172 (41)	234 (42)	248 (43)	425 (37)	265 (46)
Missing	9 (0)	1 (0)	0 (0)	7 (1)	1 (0)
**BMI**					
*Median [IQR]*	*19.5 [17.5–22.0]*	*19.6 [17.9–22.2]*	*19.8 [17.9–22.2]*	*19.2 [17.2–21.7]*	*19.8 [17.4–22.1]*
<18.5	885 (36)	173 (33)	172 (33)	377 (40)	163 (36)
≥18.5	1552 (64)	349 (67)	352 (67)	555 (60)	296 (64)
Missing	448 (16)	39 (7)	59 (10)	232 (20)	118 (21)
**Concomitant TB^2^**					
Yes	183 (7)	20 (4)	20 (4)	104 (10)	39 (8)
No	2338 (93)	503 (96)	495 (96)	906 (90)	434 (92)
Missing	364 (13)	38 (7)	68 (12)	154 (13)	104 (18)
**WHO stage**					
I–II	1504 (59)	355 (69)	339 (64)	530 (52)	280 (59)
III–IV	1041 (41)	163 (31)	190 (36)	491 (48)	197 (41)
Missing	340 (12)	43 (8)	54 (9)	143 (12)	100 (17)
**Site**					
Hospital	1217 (42)	187 (33)	258 (44)	538 (46)	234 (41)
Health Centre	1668 (58)	374 (67)	325 (56)	626 (54)	343 (59)
**Year of diagnosis**					
2015	420 (15)	72 (13)	65 (11)	200 (17)	83 (14)
2016	859 (30)	115 (20)	269 (46)	318 (27)	157 (27)
2017	947 (33)	225 (40)	173 (30)	368 (32)	181 (31)
2018	659 (23)	149 (27)	76 (13)	278 (24)	156 (27)

^1^Percentages calculated after exclusion of participants with missing data for the respective variables.

**^2^**Persons receiving treatment for active TB were considered to have concomitant TB.

### Cascade of care

Initiation of ART was registered for 2476 participants (86%). Compared with persons diagnosed through VCT, participants diagnosed through community-based testing or PICT were less likely to initiate ART (adjusted odds ratio [AOR] 0.44, 95% confidence interval [CI] 0.29–0.66 and AOR 0.65, 95% CI 0.44–0.97, respectively), as were those with late WHO stage at HIV diagnosis (AOR 0.67, 95% CI 0.52–0.87; Table 2).

**Table 2.** Uni- and multivariable analysis of factors associated with ART initiation

	ART initiation	No ART initiation	Univariate analysis			Multivariable analysis		
	n (%)	n (%)	OR	95% CI	p	AOR	95% CI	p
n = 2885	2476 (86)	409 (14)						
**Age (years)**								
16–25	477 (84)	89 (16)	0.76	0.57–1.02	0.07	**		
26–35	1050 (86)	175 (14)	0.85	0.67–1.09	0.21			
≥36	906 (88)	129 (12)	ref					
**Gender**								
Female	1487 (87)	217 (13)	ref					
Male	989 (85)	183 (16)	0.79	0.64–0.98	0.03	**		
**BMI**								
<18.5	779 (88)	106 (12)	0.86	0.66–1.11	0.24	*		
≥18.5	1390 (90)	162 (10)	ref					
**TB**								
Yes	163 (89)	20 (11)	0.95	0.59–1.54	0.83	*		
No	2094 (90)	244 (10)	ref					
**WHO stage**								
I–II	1372 (91)	132 (9)	ref					
III–IV	910 (87)	131 (13)	0.67	0.52–0.86	0.002	0.67	0.52–0.87	0.003
**Test modality**								
VCT	509 (91)	52 (9)	ref					
Community	479 (82)	104 (18)	0.47	0.33–0.67	<0.001	0.44	0.29–0.66	<0.001
PICT	979 (84)	185 (16)	0.54	0.39–0.75	0.0002	0.65	0.44–0.97	0.035
Other	509 (88)	68 (12)	0.76	0.52–1.12	0.17	0.82	0.51–1.32	0.42

*Not included in multivariable analysis due to p > 0.2 in univariate analysis.

**Excluded in multivariate analysis with forward removal.

Of the 2476 participants who started ART, 2043 were included in the analysis of retention in care (Figure 1). Among these, 1422 (70%) remained in care 12 months after ART initiation. Among those not remaining in care, 128/621 (21%) were registered as dead and 493/621 (79%) were lost to follow-up. The following variables were independently associated with lack of retention in care: age 16–25 years (AOR 0.62, 95% CI 0.45–0.85), male gender (AOR 0.77, 95% CI 0.62–0.96) and BMI <18.5 (AOR 0.75, 95% CI 0.61–0.94). Type of test modality was not associated with retention in care (Table 3).

**Table 3.** Uni- and multivariable analysis of factors associated with retention in care

	Retention	Lack of retention	Univariate analysis			Multivariable analysis		
	n (%)	n (%)	OR	95% CI	p	AOR	95% CI	p
n = 2043	1422 (70)	621 (30)						
**Age**								
16–25	203 (64)	112 (36)	0.72	0.54–0.94	0.02	0.62	0.45–0.85	0.003
26–35	598 (69)	265 (31)	0.89	0.72–1.10	0.29	0.81	0.64–1.03	0.09
≥36	594 (72)	235 (28)	ref			ref		
**Gender**								
Female	814 (71)	331 (29)	ref			ref		
Male	608 (68)	290 (32)	0.80	0.66–0.97	0.02	0.77	0.62–0.96	0.02
**BMI**								
<18.5	468 (67)	229 (33)	0.71	0.58–0.87	0.001	0.75	0.61–0.94	0.01
≥18.5	861 (74)	300 (26)	ref			ref		
**TB**								
Yes	98 (65)	52 (35)	0.77	0.54–1.09	0.14	*		
No	1234 (71)	504 (29)	ref					
**WHO stage**								
I–II	778 (72)	297 (28)	ref					
III–IV	556 (68)	266 (32)	0.80	0.65–0.97	0.03	*		
**Test modality**								
VCT	348 (75)	119 (25)	ref					
Community	291 (68)	134 (32)	0.74	0.55–0.99	0.05	*		
PICT	474 (68)	227 (32)	0.71	0.55–0.93	0.01			
Other	309 (69)	141 (31)	0.75	0.56–1.00	0.05			

*Excluded in multivariable analysis with forward removal.

Among 1806 participants with ART duration >6 months, VL results recorded during the first 12 months after starting ART were available for 1046 (58%). Participants with available VL results were more likely to have been diagnosed through VCT and during the latter years of the study period (Table 4). Among these 1046 ART recipients, 953 (91%) met criteria for virological suppression. No association with test modality, or with other variables, was observed in uni- or multivariable analyses (Table 4).

**Table 4.** Viral load result available, among those eligible for testing (ie in care at time points for routine viral load testing, at 6 and 12 months after ART initiation)

	No viral load result	Viral load result
Total N (%)	760	1046
**Age (years)**		
16–25	139 (46)	164 (54)
26–35	338 (44)	432 (56)
≥36	275 (39)	425 (61)
**Gender**		
Female	445 (42)	622 (58)
Male	315 (43)	424 (57)
**BMI**		
<18.5	248 (44)	316 (56)
≥18.5	435 (40)	640 (60)
**Clinical stage**		
I–II	384 (38)	627 (62)
III–IV	323 (48)	348 (52)
**TB treatment**		
Yes	657 (42)	900 (58)
No	52 (43)	69 (57)
**Site**		
Hospital	359 (49)	369 (50)
Health centre	401 (37)	677 (63)
**Year of diagnosis**		
2015	142 (60)	93 (40)
2016	268 (50)	273 (50)
2017	233 (37)	398 (63)
2018	117 (29)	282 (71)
**Test modality**		
VCT	148 (36)	259 (64)
Community	172 (48)	188 (52)
PICT	289 (44)	370 (56)
Other	151 (40)	299 (60)

### Cascade of care in analyses disaggregated by sex

Results of separate analyses disaggregated by sex for the different steps in the cascade of care are presented in Table 5 and Table 6. For female participants, diagnosis through community-based testing modalities and concomitant TB infection were negatively associated with ART initiation in multivariable analysis compared with the respective reference groups (AOR 0.47, 95% CI 0.27–0.82 vs. AOR 0.39, 95% CI 0.20–0.76). Women aged 16–25 years were less likely to remain in care compared with females aged ≥36 years (AOR 0.59, 95% CI 0.40–0.86). There were no independently significant associations between test modality and outcome in analyses of retention in care and viral suppression.

**Table 5.** Uni- and multivariable analysis of factors associated with viral suppression

	Suppressed	Unsuppressed	Univariate analysis			Multivariable analysis		
	n (%)	n (%)	OR	95% CI	p	AOR	95% CI	p
n = 1046	953 (91)	93 (9)						
**Age**								
16–25	145 (88)	19 (11)	0.64	0.35–1.17	0.15	**		
26–35	393 (91)	39 (9)	0.85	0.52–1.38	0.51			
≥36	392 (92)	33 (8)	ref					
**Gender**								
Female	574 (92)	48 (8)	ref					
Male	379 (89)	45 (11)	0.70	0.46–1.08	0.11	**		
**BMI**								
<18.5	287 (91)	29 (9)	0.91	0.57–1.46	0.70	*		
≥18.5	586 (92)	54 (8)	ref					
**TB**								
Yes	61 (88)	8 (12)	0.71	0.33–1.55	0.39	*		
No	823 (91)	77 (9)	ref					
**WHO stage**								
I–II	579 (92)	48 (8)	ref					
III–IV	309 (89)	39 (11)	0.66	0.42–1.02	0.06	**		
**Test modality**								
VCT	241 (93)	18 (7)	ref					
Community	173 (92)	15 (8)	0.86	0.42–1.76	0.68	**		
PICT	329 (89)	41 (11)	0.60	0.34–1.07	0.08			
Other	210 (92)	19 (8)	0.82	0.42–1.61	0.58			

Suppressed: ≥1 VL <1000 copies/ml, with no recorded VL ≥ 1000 copies/ml. Unsuppressed: ≥1 VL ≥1000 copies/ml.

*Not included in multivariable analysis due to p > 0.2 in univariate analysis.

**Excluded in multivariate analysis with forward removal.

**Table 6.** Uni- and multivariable analysis of factors associated with each step of the cascade of care

	ART initiation	No ART initiation	Univariate analysis			Multivariable analysis		
	n (%)	n (%)	OR	95% CI	p	AOR	95% CI	p
**n = 1704** **Age (years)**	1487 (87)	217 (13)						
16–25	402 (86)	67 (14)	0.60	0.40–0.91	0.02	**		
26–35	656 (86)	103 (14)	0.64	0.43–0.94	0.02			
≥36	400 (91)	40 (9)	ref		ref			
**BMI**								
<18.5	428 (90)	46 (10)	1.05	0.72–1.52	0.8	*		
≥18.5	836 (90)	94 (10)	ref		ref			
**TB**								
Yes	61 (84)	12 (16)	0.49	0.26–0.94	0.03	0.39	0.20–0.76	0.01
No	1286 (91)	125 (9)	ref		ref			
**WHO stage**								
I–II	895 (92)	75 (8)	ref		ref	**		
III–IV	477 (88)	68 (13)	0.59	0.42–0.83	0.003			
**Test modality**								
VCT	296 (91)	30 (9)	ref		ref	ref		
Community	274 (82)	61 (18)	0.45	0.29–0.73	0.001	0.47	0.27–0.82	0.01
PICT	643 (88)	89 (12)	0.73	0.47–1.13	0.16	0.95	0.55–1.65	0.87
Other	274 (88)	37 (12)	0.75	0.45–1.25	0.26	0.93	0.48–1.81	0.93
	**Retention**	**Lack of retention**	**Univariate analysis**			**Multivariable analysis**		
	n (%)	n (%)	OR	95% CI	p	AOR	95% CI	p
**n = 1145**	814 (71)	331 (29)						
**Age (years)**								
16–25	164 (65)	89 (35)	0.65	0.46–0.93	0.02	0.59	0.40–0.86	0.01
26–35	364 (72)	140 (28)	0.92	0.68–1.26	0.63	0.91	0.66–1.28	0.60
≥36	269 (74)	96 (26)	ref		ref			
**BMI**								
<18.5	261 (69)	115 (31)	0.75	0.56–0.99	0.04	**		
≥18.5	494 (75)	163 (25)	ref		ref			
**TB**								
Yes	39 (68)	18 (32)	0.83	0.47–1.47	0.52	*		
No	725 (72)	277 (28)	ref		ref			
**WHO stage**								
I–II	468 (74)	167 (26)	ref		ref	**		
III–IV	302 (70)	130 (30)	0.83	0.63–1.09	0.18			
**Test modality**								
VCT	207 (76)	67 (25)	ref		ref	**		
Community	175 (72)	69 (28)	0.82	0.55–1.21	0.32			
PICT	273 (70)	117 (30)	0.75	0.53–1.07	0.12			
Other	159 (67)	78 (33)	0.65	0.45–0.97	0.03			
	**Suppressed**	**Unsuppressed**	**Univariate analysis**			**Multivariable analysis**		
	n (%)	n (%)	OR	95% CI	p	AOR	95% CI	p
**n = 622**	574 (92)	48 (8)						
**Age (years)**								
16–25	122 (91)	12 (9)	0.76	0.34–1.71	0.51	*		
26–35	251 (92)	21 (8)	0.89	0.45–1.82	0.77			
≥36	186 (93)	14 (7)	ref		ref			
**BMI**								
<18.5	166 (93)	12 (7)	1.09	0.54–2.21	0.8	*		
≥18.5	354 (93)	28 (7)	ref		ref			
**TB**								
Yes	21 (84)	4 (16)	0.41	0.13–1.25	0.12	**		
No	513 (93)	40 (7)	ref		ref			
**WHO stage**								
I–II	366 (93)	26 (7)	ref		ref	*		
III–IV	172 (91)	18 (10)	0.68	0.36–1.27	0.23			
**Test modality**								
VCT	150 (96)	6 (4)	ref		ref	**		
Community	99 (93)	8 (8)	0.50	1.17–1.47	0.21			
PICT	219 (89)	26 (11)	0.34	1.14–0.84	0.02			
Other	115 (94)	8 (7)	0.57	0.19–1.70	0.3			

*Not included due to univariate p > 0.2.

**Excluded in multivariable analysis.

**Table 7.** Male participants: uni- and multivariable analysis of factors associated with each step of the cascade of careresult available, among those eligible

	ART initiation	No ART initiation	Univariate analysis			Multivariable analysis		
	n (%)	n (%)	OR	95% CI	p	AOR	95% CI	p
**n = 1172**	1487 (87)	217 (13)						
**Age (years)**								
16–25	75 (77)	22 (23)	0.60	0.35–1.01	0.05	0.45	0.24–0.83	0.01
26–35	394 (85)	71 (15)	0.98	0.70–1.37	0.89	0.98	0.64–1.50	0.92
≥36	506 (85)	89 (15)	ref		ref			
**BMI**								
<18.5	351 (86)	58 (14)	0.73	0.50–1.07	0.1	**		
≥18.5	554 (89)	67 (11)	ref		ref			
**TB**								
Yes	102 (94)	7 (6)	2.13	0.96–4.69	0.06	**		
No	808 (87)	118 (13)	ref		ref			
**WHO stage**								
I–II	477 (90)	56 (11)	ref		ref	*		
III–IV	433 (89)	62 (13)	0.82	0.56–1.20	0.31			
**Test modality**								
VCT	213 (91)	21 (9)	ref		ref	ref		
Community	205 (83)	43 (17)	0.47	0.27–0.82	0.01	0.39	0.19–0.78	0.01
PICT	336 (79)	89 (21)	0.37	0.22–0.62	0.0001	0.34	0.17–0.65	0.001
Other	235 (89)	30 (11)	0.77	0.43–1.39	0.39	0.56	0.26–1.17	0.12
	**Retention**	**Lack of retention**	**Univariate analysis**			**Multivariable analysis**		
	n (%)	n (%)	OR	95% CI	p	AOR	95% CI	p
**n = 898**	608 (68)	290 (32)						
**Age (years)**								
16–25	39 (63)	23 (37)	0.73	0.42–1.26	0.25	**		
26–35	234 (65)	125 (35)	0.89	0.59–1.07	0.14			
≥36	325 (70)	139 (30)	ref		ref			
**BMI**								
<18.5	207 (65)	114 (36)	0.68	0.50–0.92	0.01	0.68	0.50–0.92	0.01
≥18.5	367 (73)	137 (27)	ref		ref			
**TB**								
Yes	59 (63)	34 (37)	0.77	0.49–1.21	0.26	*		
No	509 (69)	227 (31)	ref		ref			
**WHO stage**								
I–II	310 (71)	130 (30)	ref		ref	**		
III–IV	254 (65)	136 (35)	0.78	0.58–1.05	0.10			
**Test modality**								
VCT	141 (73)	52 (27)	ref		ref	**		
Community	116 (64)	65 (36)	0.65	0.42–1.02	0.06			
PICT	201 (65)	119 (35)	0.62	0.42–0.92	0.02			
Other	150 (70)	63 (30)	0.88	0.57–1.35	0.56			
	**Suppression**	**Unsuppressed**	**Univariate analysis**			**Multivariable analysis**		
	n (%)	n (%)	OR	95% CI	p	AOR	95% CI	p
**Age (years)**								
16–25	23 (77)	7 (23)	0.30	0.12–0.80	0.02	0.30	0.12–0.80	0.02
26–35	142 (89)	18 (11)	0.72	0.36–1.44	0.36	0.73	0.37–1.44	0.36
≥36	206 (92)	19 (8)	ref		ref			
**BMI**								
<18.5	121 (88)	17 (23)	0.80	0.41–1.53	0.50	*		
≥18.5	232 (90)	26 (10)	ref		ref			
**TB**								
Yes	40 (91)	4 (9)	1.19	0.40–3.52	0.75	*		
No	310 (89)	37 (11)	ref		ref			
**WHO stage**								
I–II	213 (91)	22 (9)	ref		ref	*		
III–IV	137 (87)	21 (13)	0.67	0.36–1.27	0.22			
**Test modality**								
VCT	91 (88)	12 (12)	ref		ref	*		
Community	74 (91)	7 (9)	1.39	0.52–3.72	0.51			
PICT	119 (89)	15 (11)	1.04	0.47–2.34	0.91			
Other	95 (90)	11 (10)	1.14	0.48–2.71	0.77			

*Not included due to univariate p > 0.2.

**Excluded in multivariable analysis.

**Figure 2.**
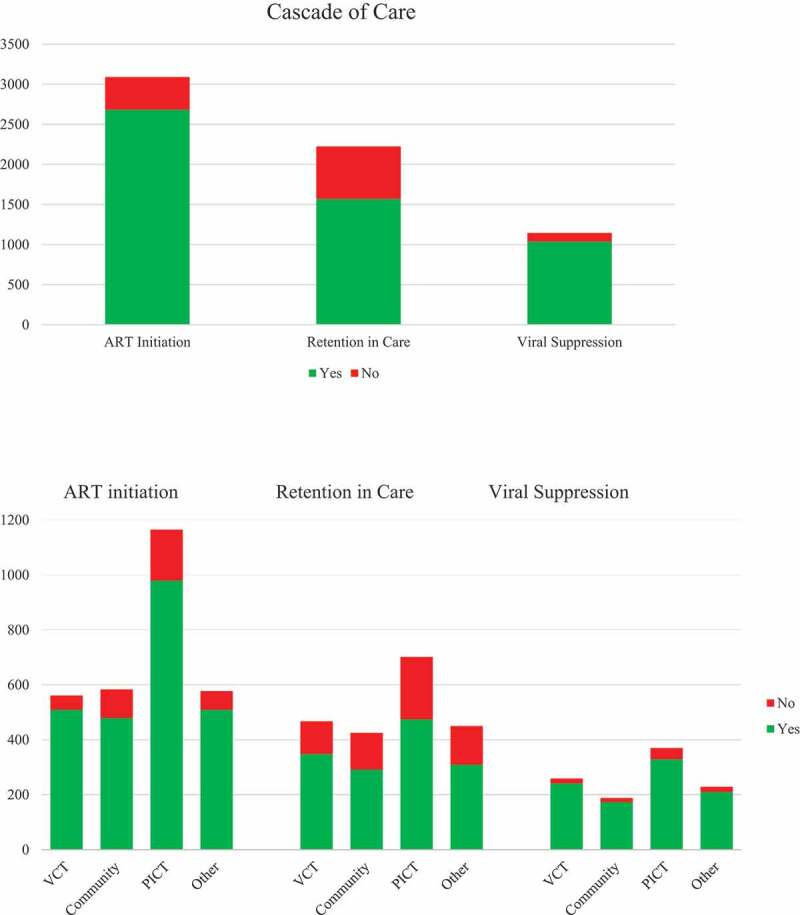
Cascade of HIV care for the overall population and disaggregated by test modality

Male participants diagnosed through community-based testing and through PICT were less likely to initiate ART compared with those diagnosed through VCT in multivariable analysis (AOR 0.39, 95% CI 0.19–0.78 vs. AOR 0.34, 95% CI 0.17–0.65, respectively). In addition, men aged 16–25 years were less likely to start ART compared with those aged ≥36 years (AOR 0.45, 95% CI 0.24–0.83). Men having BMI <18.5 were at increased risk of lack of retention in care (AOR 0.68, 95% CI 0.5–0.92). Lack of viral suppression was more common among men aged 16–25 years compared with those aged ≥36 years (AOR 0.30, 95% CI 0.12–0.80). There were no independently significant associations between test modality and outcome in analyses of retention in care and viral suppression. Figure 2 shows the cascade of care for the overall popualtion and disaggregated by test modality.

## Discussions

Similar to many other low-income countries, it is estimated that 65% of PLHIV in Ethiopia are not aware of their HIV status, despite scale-up of HIV services during the last decade [2]. Delayed HIV diagnosis is reflected by late presentation to care [17], which leads to worse prognosis for the individual and allows for unrecognized community transmission. Finding new strategies to increase access to testing, especially for populations that are not adequately reached by traditional test modalities, is therefore a priority for HIV programmes. Decentralization and community engagement are recognized as key components for achieving high HIV care coverage in areas with successful rollout of testing and treatment services [18]. Community-based testing has been shown to increase uptake of testing [3], but it is uncertain how type of test modality affects the subsequent cascade of HIV care.

In this study, persons diagnosed through community-based testing were younger and a greater proportion were men, supporting previous data that this test modality has better capacity to reach individuals with lower likelihood to present at health facilities for HIV testing [3]. In addition, and in agreement with other reports [4,5], persons diagnosed through community-based testing were less likely to have advanced disease at diagnosis.

However, diagnosis through community-based testing was associated with lower rates of ART initiation. This difference was observed both for women and for men in analyses disaggregated by sex. Several previous studies from sub-Saharan Africa have identified gaps in linkage to care in persons diagnosed through community-based testing [3]. Using VCT for reference, home-based testing has been associated with lower linkage to care in Mozambique (36% vs. 98% [4]) as well as in Tanzania (10% vs. 53% [5]). Similar findings have been reported from South Africa, where only 42% of PLHIV diagnosed through mobile testing started ART within 3 months of diagnosis [6].

Compared with these studies, the differences in linkage between facility-based and community-based testing in our study were relatively small (91% vs. 82%). Linkage to care among patients diagnosed through different types of community-based testing could depend on many factors, including target population and structure for delivery of HIV care. In this study, most community-based testing was performed by national NGOs, well established in the respective communities, which could contribute to a higher proportion of ART initiation. Some previous studies on this topic were conducted before the introduction of universal ART, and linkage to care was assessed by availability of CD4 results [4]. It is likely that current guidelines, recommending a ‘test-and-treat’ strategy, provide better motivation to engage in care for newly diagnosed PLHIV.

Further efforts to improve linkage to care for persons diagnosed through community-based testing are needed to increase completion rates of the HIV care cascade. Several interventions for facilitated linkage to ART initiation have been investigated, such as comprehensive care and additional tracing for individuals who do not present to care after diagnosis [19–21]. Home-based ART initiation has been explored as an option to increase linkage to care among persons diagnosed through community testing, with promising results in a randomized controlled trial from Lesotho [22]. Direct access to HIV care and ART after provision of test results appears to be critical; in a study from Tanzania, health service provision in the facility carrying out the HIV test was identified as the most important factor for linkage to care [23].

HIV diagnosis through PICT as test modality was also associated with lower linkage to care in our study. Patients diagnosed through PICT had a more advanced disease stage at diagnosis, whereas the opposite was found for those diagnosed through community-based testing. Various characteristics of individuals (including some for which we did not have information, such as socio-economic factors) could be involved in differences observed between test modality and linkage to care [24]. In sex-disaggregated analysis, we identified gaps in linkage to care not related to testing modality that merit further attention. Among women, those with concomitant TB were less likely to initiate ART, suggesting worse outcomes among women with TB/HIV coinfection. In addition, men aged 16–25 years had higher likelihood of not starting ART, implying the need of further focus on this category in HIV programmes.

Overall, retention in care was unsatisfactory in our study population, with <70% remaining in care at 12 months after starting ART. This finding is in accordance with other reports from Ethiopia, as well as from other sub-Saharan African countries [25], illustrating a major problem in HIV care provision. In most cases, attrition from care was attributed to loss to follow-up. This phenomenon has been found to have heterogeneous causes in Ethiopia, including unrecognized mortality, self-transfer of care and seeking of alternative therapies [26]. Our study design did not allow for tracing to investigate reasons for loss to follow-up. Yet, retention in care did not show statistically significant differences with regard to test modality. Men, as well as persons aged 16–25 years, were less likely to remain in care, which is in agreement with previous studies from sub-Saharan Africa [12,27]. One possible reason for high rates of attrition among men can be more advanced disease at diagnosis [15]. This is supported by the association between malnutrition and lack of retention in care among men in our study, which suggests unrecognized mortality as an important cause of attrition from care. Other factors that have been implied are work mobility and lack of clinics offering care adapted to male clients [28]. Among women, those aged 16–25 years were less likely to remain in care. Lower retention in care among adolescents has been reported from sub-Saharan Africa [29], and diverse reasons for this phenomenon are probably involved. However, community-based strategies and HIV services specifically adapted to young persons have been shown to increase retention in care among adolescents [30].

Viral suppression was registered for 91% of participants with available VL data. This rate is in agreement with previous studies from this uptake area [31] and suggests overall satisfactory virological outcomes among persons on ART. However, VL results were missing for more than one-third of persons starting ART. During the study period, VL testing was scaled up in Ethiopia following revision of guidelines recommending universal regular VL testing for all ART recipients. As a consequence, the proportion of study participants with available VL results increased over the study period. Yet, even in 2017, nearly 30% of ART recipients had not received VL testing, illustrating another challenge for HIV care in low-income settings.

Viral suppression was not significantly associated with testing modality, or with any of the characteristics investigated in the aggregated analysis. However, among men, those aged 16–25 years had significantly lower rates of viral suppression compared with those aged ≥36 years. Previous studies from the uptake area have found lack of viral suppression to be associated with male sex and advanced disease stage [31,32]. Other factors (such as age and socio-economic condition) have been reported to be associated with virological outcomes during ART from other settings [33,34]. Owing to the high proportion of missing VL results in our study population, it is not possible to exclude an effect of test modality on viral suppression, and further studies on this issue are required. The proportion of patients with available VL results was slightly lower for those diagnosed through community-based testing compared with VCT, a result that could motivate further studies on this subject.

To our knowledge, the association between test modality and the cascade of HIV care has not previously been investigated in Ethiopia. Our study was conducted in a mainly urban and semi-urban area, located along a major transport highway, which has among the highest rates of HIV infection in the country. The study participants were identified from routine care registers at public health facilities, and most were diagnosed after the introduction of ‘test-and-treat’ guidelines for universal ART in Ethiopia.

This study has certain limitations. Owing to the retrospective design, data were missing for some potential participants and they were excluded for this reason. Women diagnosed through ANC were excluded from the analysis of retention in care since these women are routinely managed in ANC clinics during pregnancy and post-partum. Furthermore, the proportion of missing VL results was high, and the analysis of associations between test modality and viral suppression has to be interpreted with care.

Participants for the study were identified at health facilities, and we did not have access to registers from community-based testing sites. Consequently, it is possible that some persons may have been lost directly after testing, which could have led to a falsely high proportion of ART initiation for clients diagnosed through community-based testing.

Regardless of underlying reasons for differences in treatment outcome, understanding of differences for clients entering HIV care from different testing modalities can enable targeted interventions, linked to testing programmes, in order to improve treatment outcomes.

## Conclusions

Individuals diagnosed with HIV through community-based testing in Ethiopia were less likely to initiate ART than persons diagnosed through VCT. However, rates of retention in care and viral suppression were similar during the first year after ART initiation with regard to type of test modality. These findings imply the need for interventions to facilitate linkage to care in community-based HIV testing programmes, but also indicate that community-based HIV case-finding results in satisfactory outcomes of HIV care.

## Paper context

Community-based HIV testing can reach populations less likely to present for facility-based testing, but it is uncertain how type of test modality influences HIV care outcomes. We studied the relationship between test modality and treatment outcomes for persons newly diagnosed with HIV in Ethiopia. Persons diagnosed through community-based testing had lower rates of ART initiation, but subsequent treatment outcomes were non-inferior, suggesting satisfactory outcomes for persons diagnosed through community-based testing if linkage to care is established.
